# Acupuncture improves depressive-like behaviors in CUMS rats by modulating lateral habenula synaptic plasticity via the BDNF/ERK/mTOR pathway

**DOI:** 10.1186/s13041-025-01247-1

**Published:** 2025-10-08

**Authors:** Simin Yan, Jia Liu, Tiansheng Zhang, Jianguo Li, Jingyu Zeng, Meng Li, Muhammad Shahzad Aslam, Junliang Shen, Tao Tong, Zhuoran You, Siyu Liu, Peng Li, Jingxuan Li, Kaiyue Gong, Simiao Wei, Chongyao Hao, Xianjun Meng

**Affiliations:** 1 Shenzhen Research Institute of Xiamen University, Shenzhen, People’s Republic of China; 2 Department of Traditional Chinese Medicine, School of Medicine, Xiamen University, Xiamen, Fujian People’s Republic of China; 3 The Fifth Hospital of Xiamen, Xiamen, Fujian People’s Republic of China; 4 Shanxi Acupuncture and Moxibustion Hospital, Taiyuan, Shanxi People’s Republic of China; 5 Affiliated Hospital of Hebei University of Engineering, Hebei, People’s Republic of China; 6 Second Clinical College, Shanxi University of Traditional Chinese Medicine, Taiyuan, Shanxi People’s Republic of China; 7 School of Traditional Chinese Medicine, Xiamen University Malaysia, 43900 Sepang, Selangor Malaysia; 8 Longyan Hospital of Traditional Chinese Medicine Affiliated to Xiamen University, Longyan, Fujian People’s Republic of China; 9 Department of Clinical Medicine, Xiamen Medical College, Xiamen, People’s Republic of China

**Keywords:** Depression, Acupuncture, Chronic unpredictable mild stress, Synaptic plasticity, BDNF/ERK/mTOR signaling pathway

## Abstract

Acupuncture has been found to alleviate depressive behaviors caused by chronic unpredictable mild stress (CUMS) in rats. This study explores how acupuncture improves depressive behaviors by modulating synaptic plasticity in the lateral habenula through stimulation of Fengfu and Shangxing acupoints. Male Sprague–Dawley rats were divided into six groups, with the control group excluded. Undergoing a 28-day CUMS protocol, the intervention groups included sham needle stimulation, daily stimulation at the Fengfu (GV16) and Shangxing (GV23) acupoints on alternate days, fluoxetine (2.1 mg/kg, 0.21 mg/mL), or electroacupuncture treatment. All rats were weighed and subjected to behavioral tests. Western blotting was used to examine the expression of the BDNF/ERK/mTOR signaling pathway and associated proteins in the lateral habenula. The monoamine neurotransmitters in serum were measured using ELISA kits. Immunofluorescence staining was used to determine the expression levels of BDNF, TrkB, SYP, and PSD95 in the lateral habenula. Golgi staining was employed to quantify dendritic spine morphology. The study showed that CUMS led to depressive-like behaviors and downregulated the BDNF/ERK/mTOR signaling pathway in the lateral habenula. It also resulted in reduced expression of monoamine neurotransmitters in peripheral blood and changes in dendritic spine length and density. Importantly, both fluoxetine and acupuncture had varying degrees of preventive and restorative effects on these changes. The findings of this study suggest that acupuncture has the potential to activate the BDNF/ERK/mTOR signaling pathway in the lateral habenula of CUMS rats, thereby enhancing synaptic plasticity and exerting an antidepressant effect.

## Introduction

Depression (also known as depressive disorder) is a prevalent mental illness that affects a significant portion of the population. According to the World Health Organization, depression affects over 300 million individuals worldwide, with an adult prevalence rate of approximately 5%. It is projected that by 2030, depression will emerge as the leading cause of global disease burden [1]. Individuals suffering from depression often exhibit symptoms such as persistent sadness, anhedonia, insomnia, and, in severe cases, self-harm and suicide ideation. These manifestations impose substantial psychological and socioeconomic burdens on families and society [2]. Currently, the treatment landscape for depression primarily revolves around selective serotonin reuptake inhibitors (SSRIs) and other antidepressant medications like fluoxetine [3]. The mechanism of action for these drugs predominantly involves inhibiting the activity of the 5-HT transporter to elevate serotonin levels in order to elicit an antidepressant effect [4]. However, these pharmacotherapies are associated with various side effects, including low response rates, prolonged onset time, and high relapse rates [5]. Consequently, it becomes imperative to explore reliable alternative therapeutic approaches.

Acupuncture, an integral component of traditional Chinese medicine, offers advantages of cost-effectiveness and minimal side effects compared to pharmacotherapy [6]. With a history spanning over 3000 years in China and other Asian countries, it has been extensively utilized for treating various ailments [7]. Electroacupuncture (EA), a treatment modality that combines acupuncture techniques with electrical stimulation to enhance therapeutic outcomes [8]. Clinical studies have demonstrated that EA is more efficacious than SSRIs in ameliorating overall condition, anxiety, and hopelessness among depressed patients [9, 10]. However, the precise mechanism underlying the antidepressant effects of acupuncture remains elusive. Therefore, elucidating the mechanism by which acupuncture treats depression assumes paramount significance for its widespread clinical implementation.

The lateral habenular nucleus (LHb) is considered a crucial structure that facilitates communication between the brain and the monoaminergic system [11]. Additionally, it is one of the few brain regions capable of regulating both dopaminergic and 5-HT systems within the brain [12, 13]. A growing body of evidence from animal and human studies also suggests an association between aberrant LHb activity and depressive symptoms such as helplessness and anhedonia (pleasure deficit) [13]. Neuroplasticity serves as the fundamental mechanism through which neurons adapt; however, this process can become disrupted in depression. Stress can induce alterations in neuroplasticity that contribute to the development of depression [14].

The “neurotrophic dysfunction hypothesis of depression” posits that neurotrophic dysfunction is a fundamental cause of the synaptic and cerebral functional alterations associated with depression. Brain-derived neurotrophic factor (BDNF), a member of the neurotrophic factor family, has been extensively investigated and is widely distributed in the central nervous system [15]. It plays a crucial role in neuronal differentiation, growth, synapse formation, plasticity, and higher cognitive functions by binding to its receptor tyrosine kinase receptor B (TrkB) [16]. Research has demonstrated that activation of BDNF and TrkB can positively modulate the expression of numerous downstream signaling pathways involved in neuronal survival and function, including the PI3K-Akt and Raf-MEK-ERK pathways [17]. Previous experimental findings have also indicated that chronic unpredictable mild stress (CUMS) depression models in rats exhibit reduced levels of BDNF and TrkB in the lateral raphe nucleus, which can be reversed by acupuncture [18].

The extracellular signal-regulated kinase (ERK) is a subtype of the mitogen-activated protein kinase (MAPK) and plays a pivotal role in the pathogenesis, symptoms, and treatment of depression [19]. As an effector downstream of BDNF-TrkB binding, ERK can activate a cascade of protein signaling mediators, including cAMP response element-binding protein (CREB). Our previous experiments have also demonstrated that acupuncture induces CREB activation in the lateral raphe nucleus and ameliorates depressive-like behavior in rats [18]. Furthermore, experimental findings indicate that acupuncture exerts antidepressant effects by modulating the ERK pathway in the hippocampus of rats [20].

The mTOR (mammalian target of rapamycin) is a serine/threonine protein kinase that functions as a pivotal regulator of cellular metabolism, exerting its influence through modulation of protein synthesis essential for synapses, thereby facilitating cell growth and proliferation [21]. Research has demonstrated that the rapid-acting antidepressant ketamine exerts its antidepressant effects by activating mTOR and promoting dendritic growth along with an increase in synapse-related protein content [22]. As a downstream signaling pathway of ERK, mTOR facilitates the phosphorylation of its downstream effectors p70 ribosomal protein S6 kinase (p70S6K) and eukaryotic initiation factor 4E binding protein 1 (4E-BP-1), thereby stimulating synaptic protein synthesis and further enhancing regeneration of neurons within the central nervous system as well as peripheral nerve axons [23].

Dendrites serve as crucial sites for the reception and transmission of neuronal information from other synapses, with their function primarily reliant on dendritic growth and the formation of dendritic spines [24]. Synaptophysin (SYP), a protein found in synaptic vesicles, can be employed as a marker to detect synaptic activity and assess synapse density and distribution [25, 26]. Postsynaptic density protein 95 (PSD-95) is predominantly localized in the postsynaptic membrane and plays a pivotal role in regulating the size and morphology of dendritic spines [27]. Consequently, alterations in these proteins may reflect changes occurring within neuronal dendritic spines and synaptic plasticity.

## Materials and methods

### Animals and groups

Sprague–Dawley (SD) male rats (100–120 g, 3–4 weeks of age) of specific-pathogen-free (SPF) grade were obtained from Beijing Huafukang Biotechnology Co., Ltd. [Animal Certificate No. SCXK (Beijing) 2019–0008]. The rats were kept in a room with controlled humidity (55 ± 5%) and temperature (23 ± 2 °C) and were exposed to a 12-h cycle of light (8:00–20:00) and darkness (20:00–8:00 the following day). Free access to appropriate food and water was offered. All operations on rats were confirmed with ethical regulations and the ethics committee of animal care of the Xiamen University, Xiamen, China (License No. XMULAC202110062). This study was conducted per the ARRIVE 2.0 guidelines for animal research, ensuring rigorous methodological standards and transparency in reporting animal experiments.

To ensure that the baseline levels were the same after a week of adaptation, we monitored the rats’ body weight weekly during the experiment and carried out a sucrose preference test after the adaptation period. The rats were then randomly divided into six groups (n = 10/group): control group (CON), CUMS group (CUMS), CUMS with fluoxetine treatment group (FLX), CUMS with acupuncture treatment group (AP), CUMS with sham-AP treatment group (Sham-AP), and CUMS with electroacupuncture treatment group (EA).

### CUMS model procedure

With a few minor changes, the CUMS rat model was created using the findings of an earlier study [28]. After 1 week of adaptation, all experimental rats with the exception of control rats randomly received 2 of 8 chronic stresses every day, and the stress methods were not repeated within 3 days. The entire stimulation process lasts 28 days. These stress programmes included strobe stimulation (12 h), alcohol odor (10 min), wet bedding (24 h), tail clamping (3 min), reversed light/dark cycle (7:00–19:00 dark, 19:00-next day 7:00 light), water deprivation (24 h), fasting (24 h), restraint stress (6 h), Crowding stimulation (6–7 rats per cage) (24 h) and no bedding (24 h).

### Interventions

All treatments in this experiment were administered daily, one hour before the commencement of the CUMS regimen, and continued for a period of 4 weeks. Fluoxetine, dissolved in double—distilled water, served as the positive control drug. Rats in the FLX group received intragastric administration of fluoxetine (2.1 mg/kg, 0.21 mg/ml, PHR1394-1G, Sigma-Aldrich, USA) for the duration of four weeks. Acupuncture was performed at the Fengfu (GV16) and Shangxing (GV23) acupoints based on our previous study [18]. GV16 is located at the posterior occipital ridge atlas joint, while GV23 is positioned at the midpoint of the rat’s anterior coronal suture. Sterilized disposable stainless steel needles (0.25 mm diameter, HanYi, Changchun, China) were inserted into both points to a depth of 5 mm at an oblique angle of 15° for 20 min. To ensure needle stability during rat needling, unique rat immobilization equipment was utilized. Rats in the AP group received acupuncture treatments every other day. For the Sham-AP group, after undergoing similar immobilization, needles were inserted 3 mm away from the GV16 and GV23 points using the same acupuncture method, depth, angle, and duration as in the acupuncture group. In the EA group, following the same immobilization and acupuncture procedure as the AP group, rats were connected to an EA apparatus (Huatuo brand electroacupuncture instrument SDZ-II). The positive pole was connected to GV16 and the negative pole to GV23, applying a dense wave current of 0.5 mA at 2 Hz frequency for 20 min. Rats in both the Sham-AP and EA groups received treatments every other day.To ensure consistency, the same restraint procedure was applied to both the model and fluoxetine groups to minimize variability induced by handling.

### Behavioral tests

The rats were weighed at consistent time intervals on days 0, 7, 14, 21, 28, and 36 throughout the duration of the experiment. In addition to weight measurements, behavioral tests were conducted during days 29–35 of the study.

#### Open field test (OFT)

The open field test was employed to assess the locomotor activity as well as anxiety- and depression-like behaviors in rats. This assessment was conducted on the 29th day of the experimental period. The apparatus utilized for this purpose comprised a black, open square box measuring 100 cm × 100 cm × 40 cm. The floor of the box was divided into an average of 4 × 4 squares. Rats were placed at the center of these 16 squares and allowed to freely explore for a duration of 5 min. A high-resolution infrared camera and Smart3.0 software was employed to record and analyze the ratio of time spent and distance traveled by each rat within the central area relative to their total time spent and distance traveled during testing sessions. Following each trial, thorough cleaning with 75% alcohol solution was performed on the square box to prevent any potential carryover effects.

#### Elevated plus maze test (EPM)

The Elevated Prison Maze (EPM) is commonly employed for the assessment of exploratory and anxiety-like behaviors in rodents. This assessment was conducted on the 30th day of the experimental period. The EPM comprises two vertical open arms (50 cm × 10 cm) and two vertical closed arms (50 cm × 10 cm × 40 cm), with a central area measuring 10 cm × 10 cm positioned in the middle, while the entire structure is elevated 80 cm above ground level. Prior to testing, each rat was placed in the central area of the cross maze, facing the open arm, and allowed to freely explore for a duration of 5 min. An infrared lamp equipped high-resolution camera was installed above the apparatus to capture and record both distance traveled by and time spent by rats in both open and closed arms. Subsequently, collected data were analyzed using Smart3.0 software. Following rhe completion of each rat’s test session, thorough cleaning and drying of the device with 75% ethanol were performed to eliminate any residual odor that could potentially impact subsequent subjects.

#### Forced swimming test (FST)

The forced swimming test (FST) is usually used to assess the degree of despair in rats. This behavioral study was conducted on the 31st day of the experiment. All rats swam in a transparent cylindrical tube for 6 min. The tube has a diameter of 0.2 m and a height of 0.45 m, with tap water filling it to a depth of 0.3 m at a temperature of 22 ± 2 °C. Before the formal test, the rats were allowed to adjust for 1 min, and then they were given a 5-min swimming test. The immobility time of the rats was recorded and analyzed using Smart 3.0 software. After each rat was tested, tap water in the plexiglass bottle was replaced to ensure the accuracy of the experiment and eliminate the influence of the previous rat.

#### Sucrose preference test (SPT)

We conducted sucrose preference tests (SPT) before and after the intervention, which are commonly employed to assess anhedonia, a cardinal symptom of depression. The post-intervention sucrose preference tests were performed on days 32–35 of the experiment. During the first 24-h period, two water bottles containing a 1% sucrose solution were placed in each rat cage. In the subsequent 24 h, each rat was exposed to one bottle of water and one bottle of a 1% sucrose solution. After another 24-h period of food and water deprivation, each rat was individually housed for the formal test. Each rat received access to both a bottle of water and a bottle of a 1% sucrose solution. After a duration of 12 h, liquid consumption was measured by weight. Sucrose preference was calculated using the following formula: sucrose preference = (sucrose consumption/(water consumption + sucrose consumption)) × 100% [29].

### Perfusion fixation and tissue acquisition

After all the modeling, therapeutic procedures and behavioral experiments, the rats were perfused and fixed, and brain tissue and serum were obtained for ELISA analysis. The rats were anesthetized using pentobarbital sodium. Blood samples were collected from the abdominal aorta to obtain serum from all rats. Three randomly selected rats from each group underwent perfusion with normal saline through the heart, followed by an injection of 4% paraformaldehyde and subsequent sacrifice. The brain tissues of these rats were extracted and stored in a 4% paraformaldehyde solution. The remaining rat brains were swiftly removed on ice. Three randomly chosen rat brains were placed in Golgi fixation solution for further analysis, specifically targeting Lateral Habenular nucleus (LHb). The LHb of the rat’s brain tissue was excised for Western blotting analysis.

### Western blotting (WB)

The lateral habenular nucleus tissue was collected and homogenized using a homogenizer in RIPA lysis buffer (R0010, Solarbio) supplemented with a protease inhibitor (04693132001, Roche) and a phosphatase inhibitor (04906837001, Roche). After 30 min of lysis on ice, the samples were centrifuged at 4 °C, and the resulting supernatant was used for protein concentration determination with a BCA protein detection kit (QB214754, Thermo Scientific).

Subsequently, protein samples were electrophoresed on a pre-prepared 10% SDS-PAGE gel and transferred to a PVDF membrane (K5NA8023B, Amersham) via electrotransfer. The PVDF membrane was then blocked with 5% skim milk powder in TBST (Tris-buffered saline with Tween 20) at room temperature for 1 h.

Following blocking, membranes were incubated overnight at 4 °C with primary antibodies: anti-BDNF (28205-1-AP, 1:1000, Proteintech), anti-TrkB (13129-1-AP, 1:2000, Proteintech), anti-ERK1/2 (11257-1-AP, 1:3000, Proteintech), anti-p-ERK1/2 (28733-1-AP, 1:2000, Proteintech), anti-mTOR (66888-1-Ig, 1:2000, Proteintech), anti-p-mTOR (2974, 1:1000, CST), anti-p70S6K (66-638-Ig, 1:2000, Proteintech), anti-p-p70S6K (AF3228, 1:1000, Affinity), anti-4E-BP1 (60246-1-Ig, 1:2000, Proteintech), anti-SYP (67864-1-Ig, 1:2000, Proteintech), and anti-PSD-95 (20665-1-AP, 1:3000, Proteintech).

After the primary antibody incubation, membranes were washed three times with TBST for 10 min each. Subsequently, the membranes were incubated with peroxidase-labeled secondary antibodies (anti-Mouse or anti-Rabbit, SA00001-1/SA00001-2, Proteintech) at room temperature for 1 h. Following another three washes with TBST, the membranes were exposed to a freshly prepared enhanced chemiluminescence (ECL) solution for 2 min and developed in a darkroom under rapid conditions.

### Enzyme-linked immunosorbent assay (ELISA)

Rat serum was collected and analyzed for levels of neurotransmitters including 5-HT (5-HT ELISA Kit, JL53170-48 T, Jianglai Biology), DA (DA ELISA Kit, E-EL-0046c, Elabscience), and NE (Rat NE ELISA Kit). ELISA assays were conducted according to the manufacturers’ protocols. Briefly, serum samples were processed, and the optical density (OD) at 450 nm was measured using an ELISA instrument (Thermo Fisher Scientific, Waltham, MA, USA).

### Immunofluorescence (IF)

Brain tissue was fixed with paraformaldehyde, paraffin-embedded, and sectioned. After antigen retrieval, the slides were washed three times in PBS (pH 7.4) for 5 min each. The sections were then blocked with BSA (GC305010, Servicebio) for 30 min. Primary antibodies were applied and incubated overnight at 4 °C. The primary antibodies used were: anti-BDNF (1:200, GB11559, Servicebio), anti-TrkB (1:500, GB11295-2, Servicebio), anti-SYP (1:500, GB15553, Servicebio), and anti-PSD95 (1:500, GB12277, Servicebio). After three washes with PBS (5 min each), the corresponding secondary antibodies were added and incubated at room temperature for 50 min in the dark. The sections were washed again three times (5 min each), followed by incubation with DAPI dye solution (G1012, Servicebio) at room temperature for 10 min in the dark. Stained tissue images were observed under an upright fluorescence microscope (200 × magnification, NIKON ECLIPSE C1, Tokyo, Japan). The ratio of positive cells was analyzed using Aipathwell software, calculated as: positive cell ratio = (number of positive cells/total number of cells) × 100% [30].

### Golgi staining

The rat brain tissue was collected and fixed in a fixation solution (G1101, Servicebio) for over 48 h. Depending on the specific region of interest, the brain tissue was sectioned into 2-3 mm thick blocks, gently washed with normal saline multiple times, and placed in a 45 ml round-bottom EP tube. Subsequently, Golgi staining solution (G1069, Servicebio) was added to fully immerse the brain tissue. The samples were kept in a cool and well-ventilated area without exposure to light for 14 days. A multi-slice scanning technique was employed to obtain comprehensive images of the brain tissue. The lateral habenula region was examined under a light microscope at 1000 × magnification (Eclipse Ci-L, Nikon, Japan). Images were captured from morphologically intact neurons using consistent illumination conditions. Image analysis was performed using Image-Pro Plus 6.0 software (Media Cybernetics, USA). For each image, a segment of the second or third order dendritic branch from a centrally located, well-stained neuron was selected. Dendritic segments ranging from 30–90 μm in length were analyzed. Within this segment, the total number of visible dendritic spines was manually counted. Dendritic spine density was calculated using the following formula:

$$ \begin{aligned} {\text{Spine}}\;{\text{density}}\left( {{\text{spines}}/10\;\upmu {\text{m}}} \right) = & {\text{Number}}\;{\text{of}}\;{\text{spines}} \\ & \quad /{\text{Dendritic}}\;{\text{length}}\left( {\upmu {\text{m}}} \right) \times 10 \\ \end{aligned} $$

### Statistical analysis

The data analysis was conducted using GraphPad Prism (version 10.0.2, GraphPad Software, San Diego, CA, USA). For normally distributed and exhibited homogenous variance data, one-way analysis of variance (ANOVA) was employed to assess between-group statistics with time or treatment as factors, followed by Dunnett multiple comparison test. All results were presented as mean ± standard deviation (x ± s), and statistical significance was defined as a *P* value < 0.05.

## Result

### The application of acupuncture demonstrates potential in ameliorating depressive-like behavior and mitigating weight gain induced by CUMS in rats

**Fig. 1:**
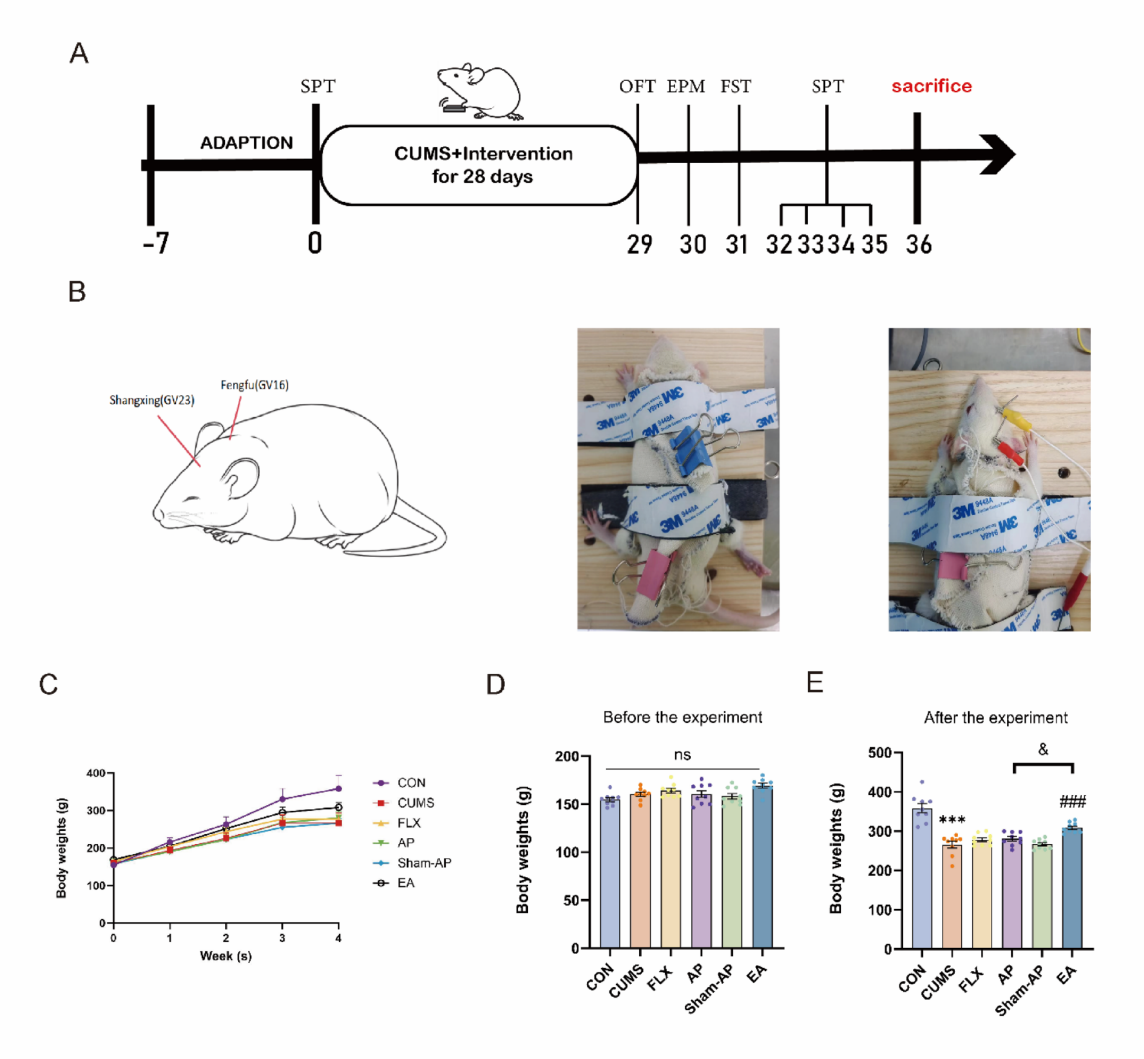
Schematic representation of experimental protocol and body weight measurement. **A** Flow chart of experiment. **B** The positioning of Fengfu (GV16) and Shangxing (GV23) acupoints, the standardization of rat fixation techniques, and the application of electroacupuncture. **C** The body weight changes of rats in 6 groups. **D** The body weight of rats on 0 days. **E** The body weight of rats after the experiment. CON: control, CUMS: chronic unpredictable mild stress, FLX: CUMS with fluoxetine treatment, AP: CUMS with acupuncture treatment, Sham-AP: CUMS with sham-AP treatment, EA: CUMS with EA treatment, the same below. Results are the mean ± standard error of the mean (x ± s), N = 10 per group. *P < 0.05, **P < 0.01, ***P < 0.001, compared to the control group. #*P* < 0.05, ##*P* < 0.01, ###*P* < 0.001, compared to the CUMS group. &*P* < 0.05, compared to the AP group

**Fig. 2:**
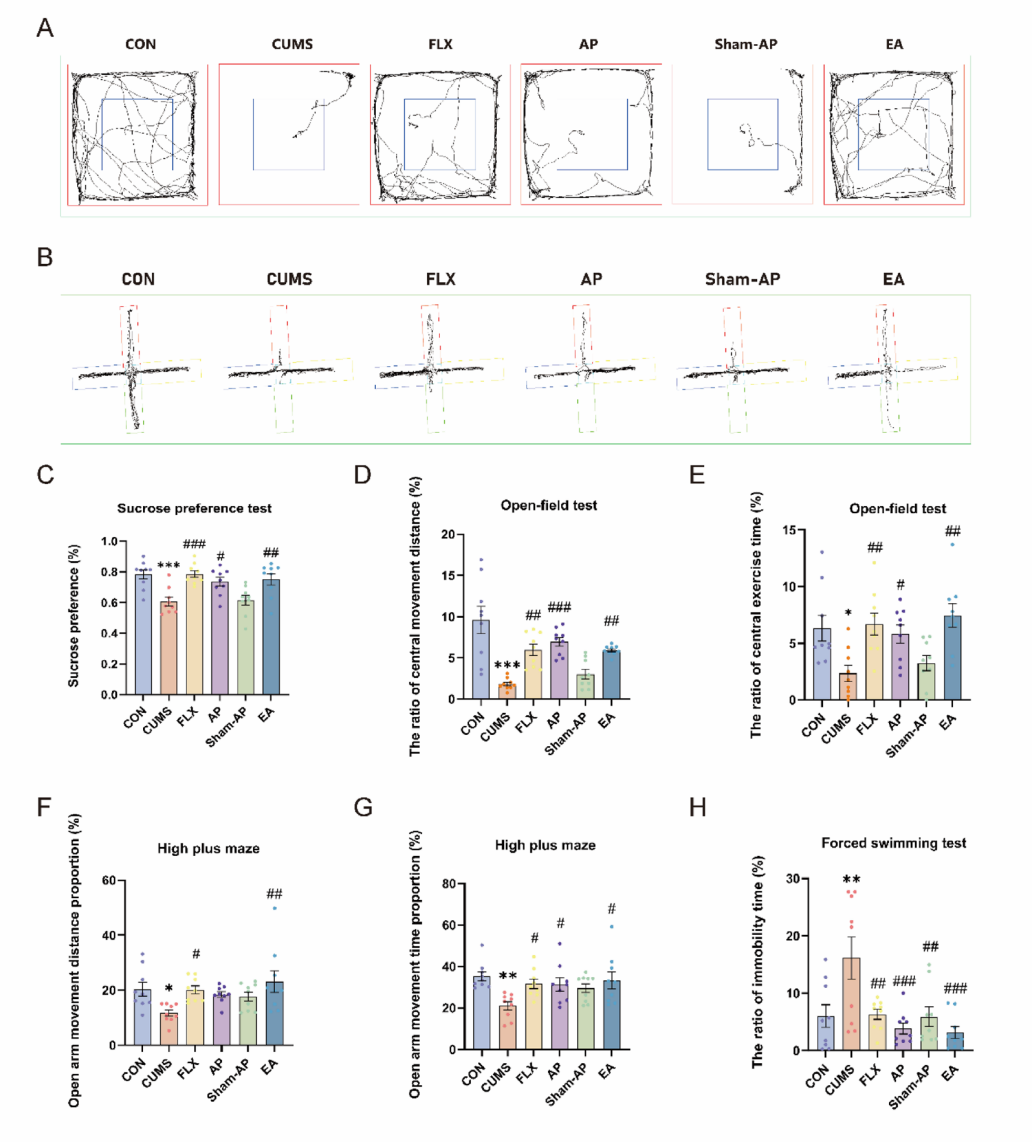
Acupuncture ameliorates depressive-behavior rats exposed to CUMS. **A**, **B** Representative tracks in EPM and in OFT. **C** The sucrose preference rate after CUMS in SPT. **D** The proportion of the distance covered by rats during central movement in OFT. **E** The proportion of time allocated to central movement by rats in OFT. **F** The proportion of time that rats spent in locomotion within the central area and the open arm of the maze in EPM. **G** The proportion of time spent by rats in the central area and the open arm relative to their total movement time in EPM. **H** The proportion of time allocated to a state of immobility. Results are the mean ± standard error of the mean (*x* ± *s*), N = 10 per group. *P < 0.05, **P < 0.01, ***P < 0.001, compared to the control group. #*P* < 0.05, ##*P* < 0.01, ###*P* < 0.001, compared to the CUMS group

The depressive-like behaviors induced by CUMS in rats were observed using behavioral experiments, and the regulatory effects of acupuncture were investigated. Following CUMS modeling, a significant decrease in rat weight was observed compared to the control group (*P* < 0.001). However, this change was reversed with EA treatment (*P* = 0.0004). There were no significant differences in rat weight between the FLX group, AP group, Sham-AP group and the CUMS group (Fig. 1C–E). From Fig. 2, compared to the control group, the CUMS group rats exhibited significant reductions in sucrose preference rate (*P* = 0.0006), central movement distance (*P* < 0.0001) and time ratio (*P* = 0.0133) in the open field test (OFT), as well as open arm movement distance (*P* = 0.0317) and time ratio (*P* = 0.0025) in the elevated plus maze (EPM). Additionally, there was a significant increase in immobility time observed in the forced swimming test (FST) (*P* = 0.0029). FLX and EA treatments significantly improved these behavioral changes caused by CUMS when compared to the CUMS group (all *P* < 0.05). AP treatment increased sucrose preference rate of rats (*P* = 0.017), central movement distance and time ratio in OFT (*P* = 0.0002, *P* = 0.0374), reduced immobility time in FST (*P* = 0.0003), and increased open arm movement time ratio in EPM (*P* = 0.0438). Sham-AP did not improve depressive-like behavior induced by CUMS in rats. Therefore, it can be concluded that fluoxetine, acupuncture, and electroacupuncture can ameliorate depressive-like behavior induced by CUMS in rats. It was observed that EA exhibited a more pronounced effect on improving body weight in CUMS rats compared to AP. However, in the behavioral assessments, no statistically significant differences were found between the effects of AP and EA on ameliorating depressive-like behaviors in CUMS rats.

### Acupuncture enhances the expression of BDNF and its receptor in the LHb, induced by CUMS in rats

**Fig. 3:**
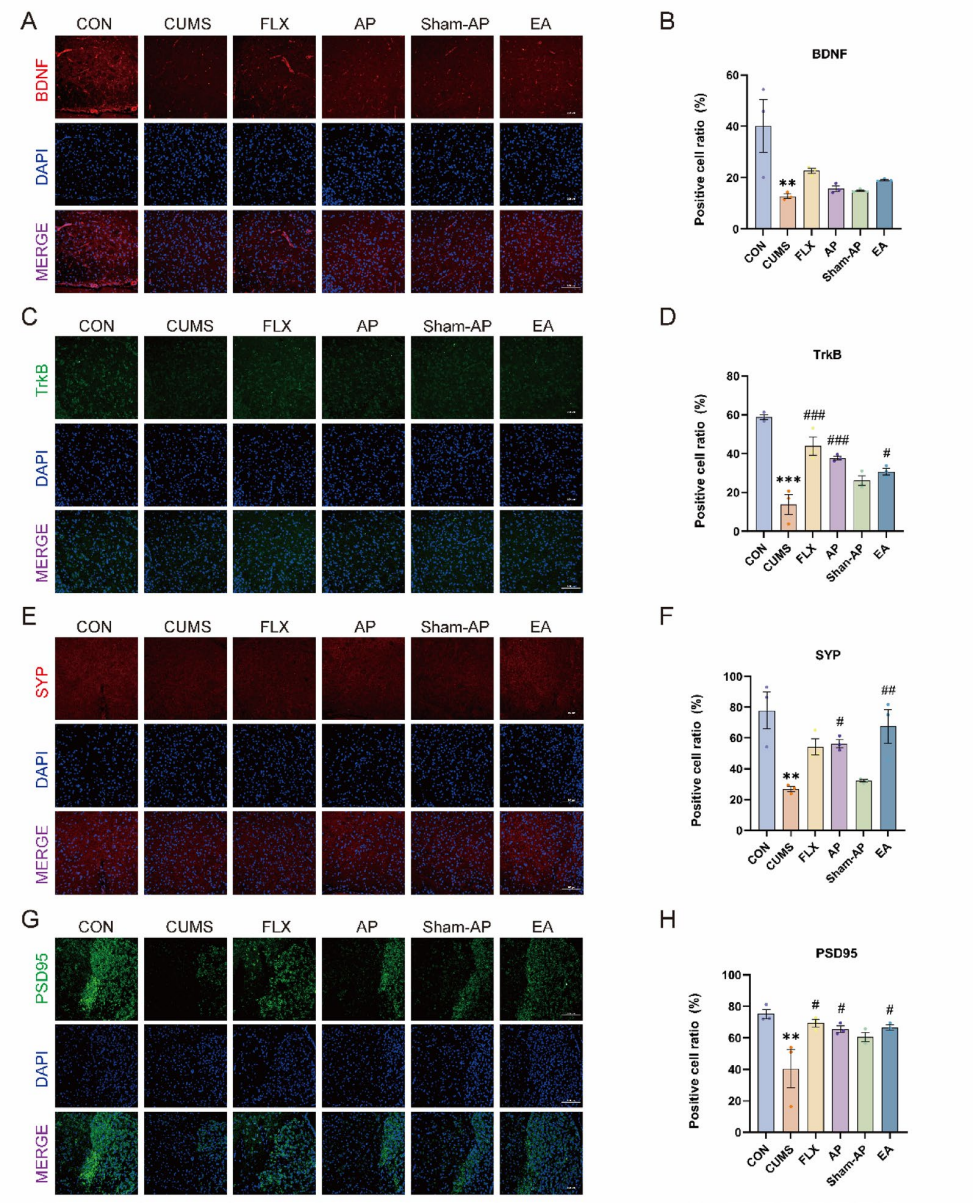
Immunofluorescence confocal microscopy imaging LHb, magnification × 200. **A**, **B** Expression of BDNF in the LHb. **C**, **D** Expression of TrkB in the LHb. **E**, **F** Expression of SYP in the LHb. **G**, **H** Expression of PSD95 in the LHb. Results are the mean ± standard error of the mean (*x* ± *s*), N = 3 per group. *P < 0.05, **P < 0.01, ***P < 0.001, compared to the control group. #*P* < 0.05, ##*P* < 0.01, ###*P* < 0.001, compared to the CUMS group

**Fig. 4:**
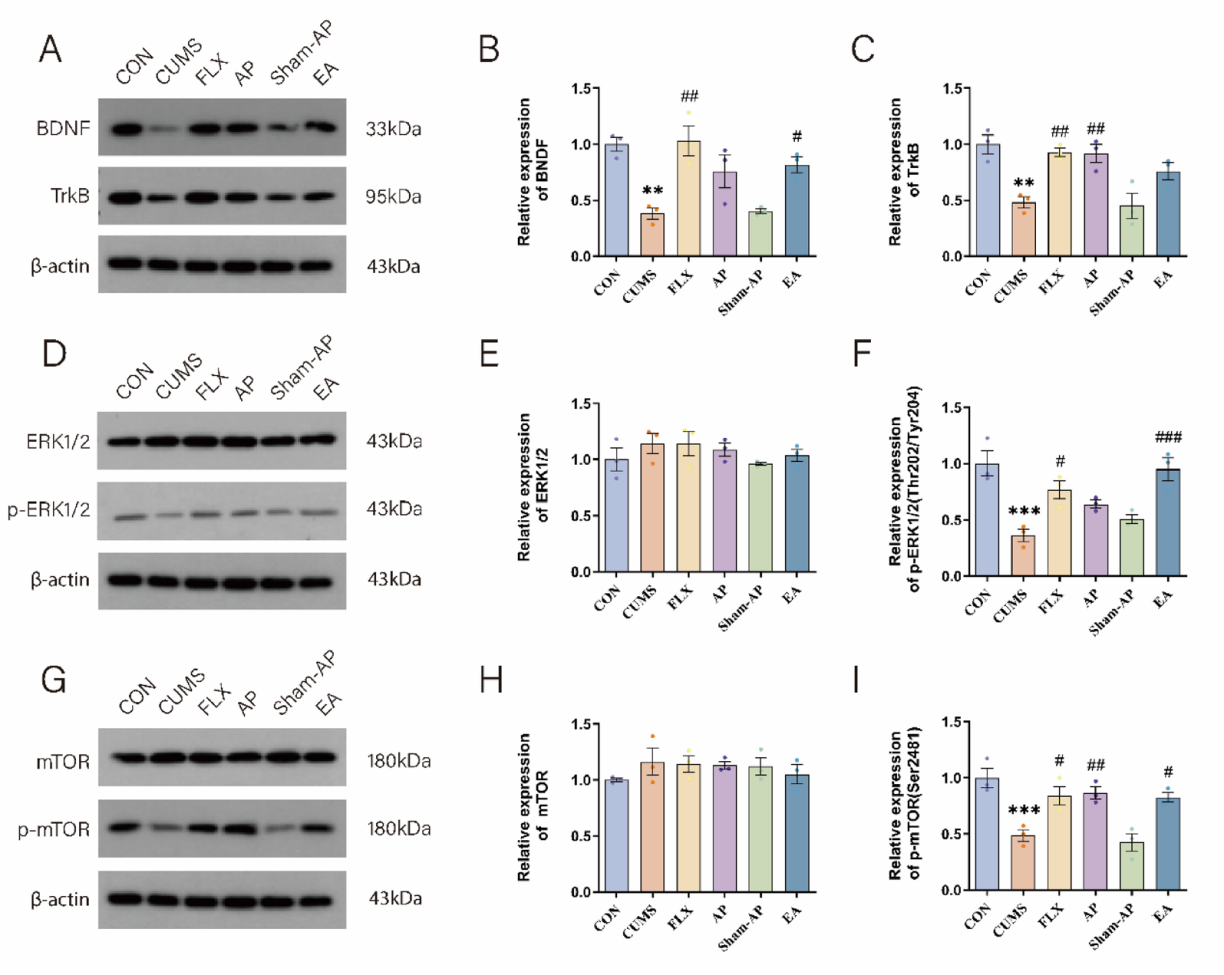
Acupuncture regulated the expression of BDNF/ERK/mTOR signaling pathway in the CUMS-induce depressed rats of LHb. **A** The expression of BDNF and TrkB protein in each group was detected by Western Blot. **B** Analysis of BDNF proteins. **C** Analysis of TrkB proteins. **D** The expression of ERK1/2 and p-ERK1/2 protein in each group was detected by Western Blot. **E** Analysis of ERK1/2 proteins. **F** Analysis of p-ERK1/2 proteins. **G** The expression of mTOR and p- mTOR protein in each group was detected by Western Blot. **H** Analysis of mTOR proteins. **I** Analysis of p-mTOR proteins. Results are the mean ± standard error of the mean (*x* ± *s*), N = 3 per group. *P < 0.05, **P < 0.01, ***P < 0.001, compared to the control group. #*P* < 0.05, ##*P* < 0.01, ###*P* < 0.001, compared to the CUMS group

From Fig. 3A–D, it is evident that the positive cell ratio of BDNF and TrkB in the CUMS group exhibited a significant decrease compared to the control group (BDNF: *P* = 0.0029; TrkB: *P* < 0.0001). There was no significant improvement in BDNF expression with any treatment, but FLX and EA treatments showed a trend towards enhancing its expression. In comparison to the CUMS group, FLX, AP, and EA all demonstrated an ability to enhance TrkB expression (FLX: *P* < 0.0001; AP: *P* = 0.0008; EA: *P* = 0.0111). The WB results (Fig. 4A–C) revealed a significant decrease in BDNF and TrkB protein expressions (BDNF: *P* = 0.002; TrkB: *P* = 0.002). FLX and EA were able to improve BDNF protein expression (FLX: *P* = 0.0014; EA: *P* = 0.0233), while FLX and AP improved TrkB protein expression (FLX: *P* = 0.0061; AP: *P* = 0.0075). However, Sham-AP did not show any improvement in BDNF and TrkB expressions induced by CUMS. No statistically significant differences were observed between the AP and EA groups in the above assessments.

### Acupuncture enhances the expression of the BDNF/ERK/mTOR signaling pathway in the LHb of rats subjected to CUMS

**Fig. 5:**
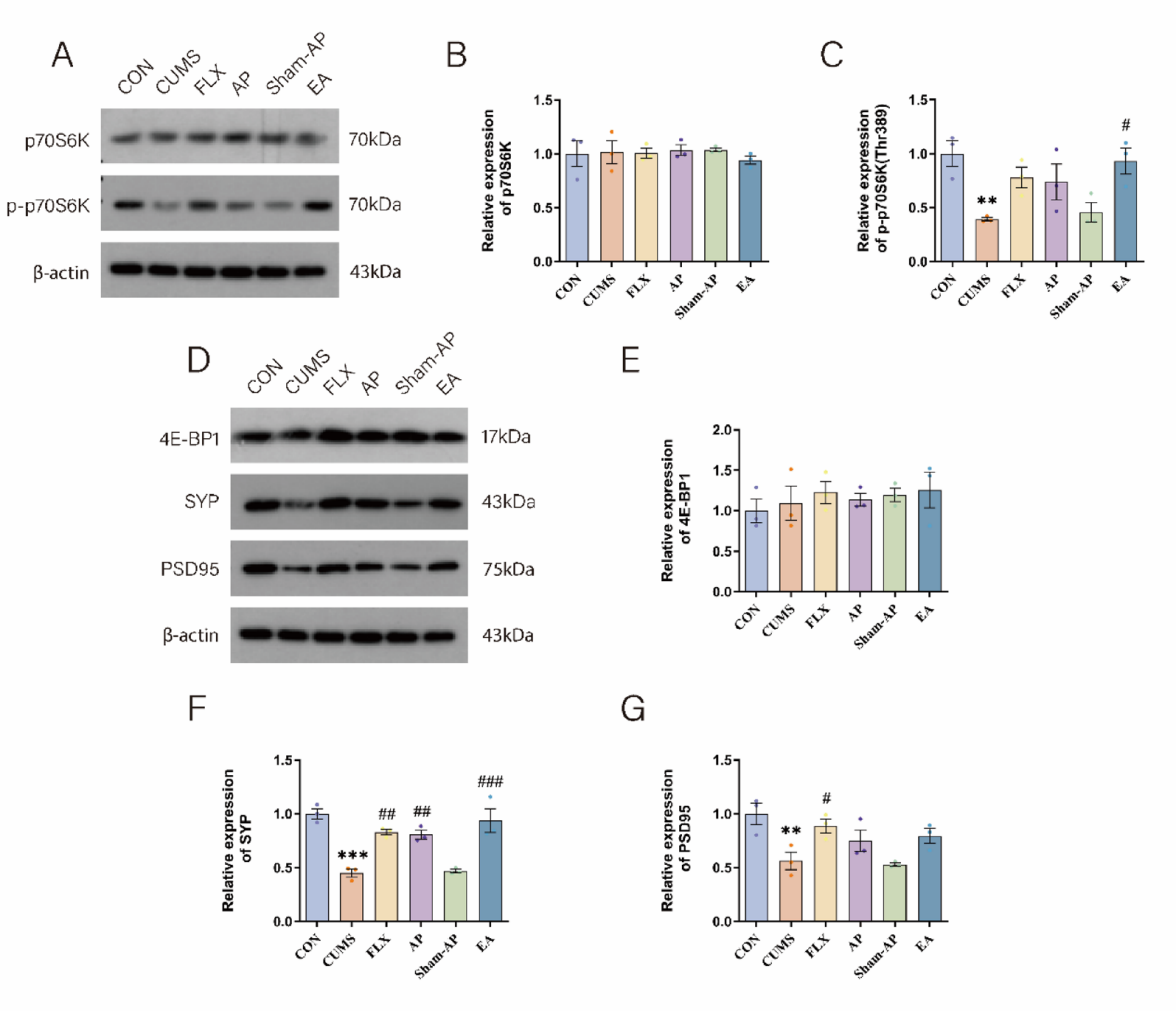
Acupuncture regulated the expression of p70S6K, p- p70S6K, 4E-BP1, SYP and PSD95 in the CUMS-induce depressed rats of LHb. **A** The expression of p70S6K and p- p70S6K protein in each group was detected by Western Blot. **B** Analysis of p70S6K proteins. **C** Analysis of p- p70S6K proteins. **D** The expression of 4E-BP1, SYP and PSD95 protein in each group was detected by Western Blot. **E** Analysis of 4E-BP1 proteins. **F** Analysis of SYP proteins. **G** Analysis of PSD95 proteins. Results are the mean ± standard error of the mean (*x* ± *s*), N = 3 per group. *P < 0.05, **P < 0.01, ***P < 0.001, compared to the control group. #*P* < 0.05, ##*P* < 0.01, ###*P* < 0.001, compared to the CUMS group

Western blotting analysis was primarily employed in this experiment to assess the expression and phosphorylation levels of the BDNF/ERK/mTOR signaling pathway in the LHb. As depicted in Fig. 4D–I, there were no significant differences in the expression levels of ERK and mTOR among the groups. However, a significant disparity was observed in terms of phosphorylation levels between these groups. Specifically, compared to the control group, there was a marked reduction in p-ERK1/2 (*P* = 0.0003) and p-mTOR (*P* = 0.0008) protein expression within the CUMS group. Conversely, treatment with FLX resulted in a significant increase in p-ERK1/2 (*P* = 0.0112) and p-mTOR (*P* = 0.0137) protein expression when compared to the CUMS group. Notably, AP administration significantly upregulated p-mTOR expression (*P* = 0.0084) and tended to increase p-ERK1/2 levels (*P* = 0.0907), while EA treatment produced more pronounced effects, significantly enhancing both p-ERK1/2 (*P* = 0.0006) and p-mTOR (*P* = 0.0173) expressions. However, there was no statistically significant difference between the AP and EA groups in terms of their effects on p-ERK1/2 and p-mTOR levels. In the Sham-AP group, there was an increase in the expression of p-ERK1/2 protein, however, these differences did not reach statistical significance. The expression of p70S6K did not significantly differ among the groups. CUMS treatment significantly reduced the level of p-p70S6K (*P* = 0.0088), which could be reversed by electrical acupuncture (*P* = 0.0191). FLX and AP also increased the expression of p-p70S6K protein but without reaching statistical significance. There were no significant differences observed in the expression of 4E-BP1 among the groups (Fig. 5A–E). Therefore, we propose that FLX, AP, and EA primarily enhance BDNF/ERK/mTOR signaling pathway activation through phosphorylation modulation within the LHb of rats.

### Acupuncture enhances the expression of proteins associated with synaptic plasticity in the ventral tegmental area of rats subjected to CUMS

Based on our previous experimental studies, we have observed that synaptic proteins SYP and PSD95 can partially reflect synaptic plasticity. From Fig. 3E–H, it is evident that CUMS significantly decreases the proportion of positive cells expressing SYP and PSD95 (SYP: *P* = 0.0011; PSD95: *P* = 0.0028). On the other hand, both AP and EA demonstrate significant improvements in the expression of SYP (AP: *P* = 0.046; EA: *P* = 0.0063), while FLX also enhances SYP expression without reaching statistical significance. However, FLX, AP, and EA all exhibit significant enhancements in PSD95 expression (FLX: *P* = 0.0105; AP: *P* = 0.025; EA: *P* = 0.0206). These findings are further supported by Western blot results shown in Fig. 5D–G where CUMS leads to a substantial reduction in protein levels of both SYP and PSD95 (SYP: *P* < 0.0001; PSD95: *P* = 0.0074). Notably, FLX treatment significantly increases SYP protein expression (*P* = 0.0016), and elevates PSD95 protein expression (*P* = 0.045). In contrast, AP treatment significantly enhances the protein expression of SYP (*P* = 0.0027), while EA treatment remarkably increases the protein level of SYP (*P* = 0.0002), but does not show statistically significant difference in terms of PSD95. No significant differences were found between the Sham-AP group and CUMS group regarding the expressions of both SYP and PSD95. Although both AP and EA were able to improve the expression of synaptic plasticity-related proteins to varying degrees, no significant differences were observed between them.

### Acupuncture enhances the abundance and density of dendritic spines in the LHb of rats subjected to CUMS

**Fig. 6:**
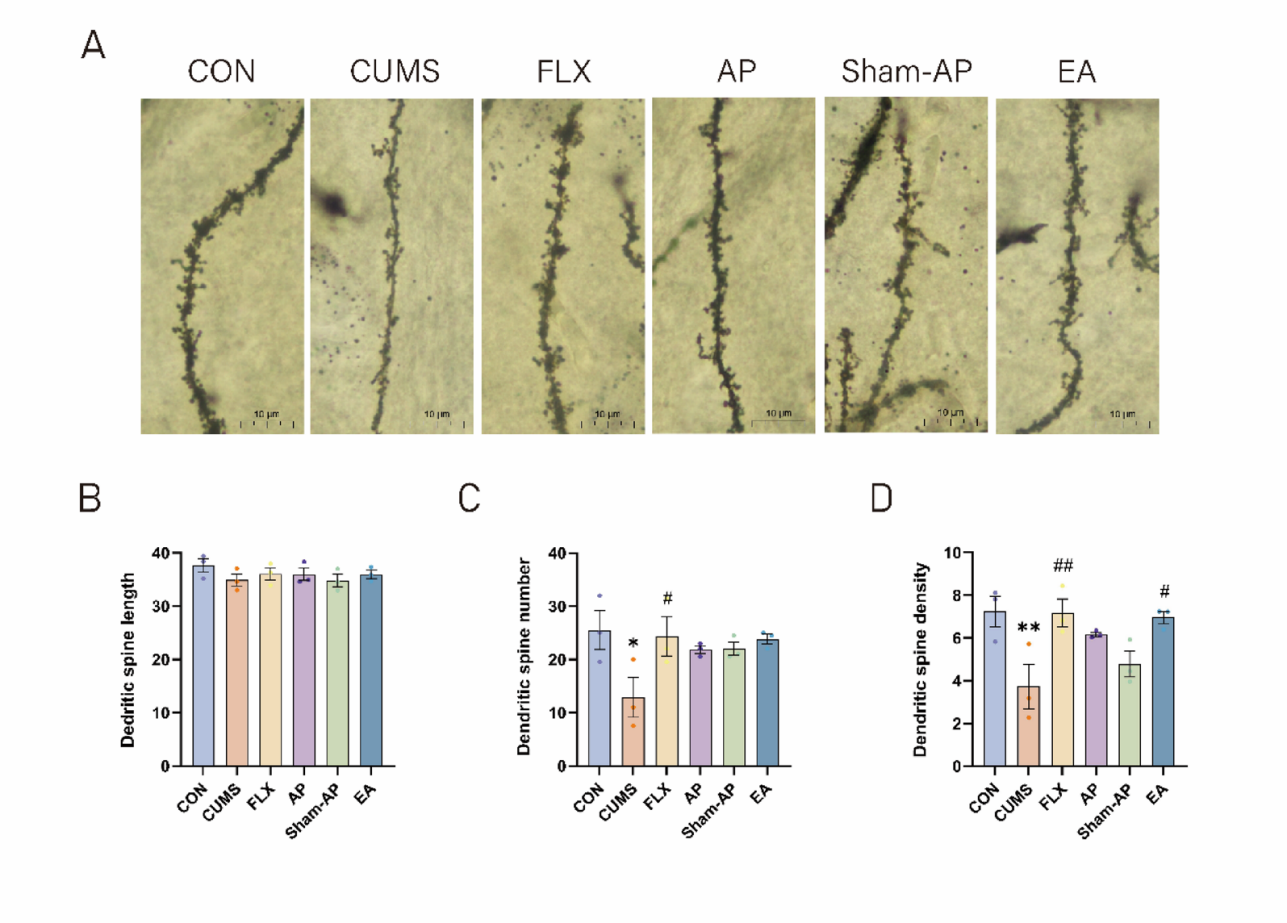
Golgi staining in the LHb. **A** Representative images of dendritic spines in the LHb of each group. Scale bar = 10 µm. **B** Quantitative analysis of the dendritic spine length in the LHb. **C** Quantitative analysis of the dendritic spine numbers in the LHb. **D** Quantitative analysis of the dendritic spine density in the LHb. Results are the mean ± standard error of the mean (*x* ± *s*), N = 3 per group. *P < 0.05, **P < 0.01, ***P < 0.001, compared to the control group. #*P* < 0.05, ##*P* < 0.01, ###*P* < 0.001, compared to the CUMS group

The morphology of dendritic spines often reflects the structural and functional characteristics of neurons, highlighting the synaptic plasticity in the brain. As shown in Fig. 6, no significant difference was observed in the length of dendritic spines among the groups. However, compared to the control group, both the number (*P* = 0.0232) and density (*P* = 0.0085) of dendritic spines were significantly reduced in the CUMS group. Notably, fluoxetine treatment effectively ameliorated this reduction in spine number (*P* = 0.04), while AP, Sham-AP, and electrical stimulation did not significantly impact spine loss. Furthermore, both fluoxetine (*P* = 0.0096) and EA (*P* = 0.015) treatments mitigated reductions in spine density. Although electroacupuncture exhibited a statistically significant improvement in dendritic spine density compared to the model group, the difference between the EA and AP groups was relatively small.

### Acupuncture has been shown to enhance the expression of monoamine neurotransmitters in rat serum

**Fig. 7:**
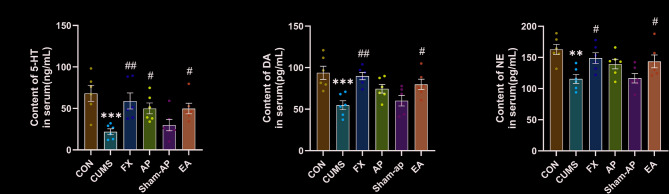
Effect of acupuncture treatment on 5-HT, DA and NE levels in serum. **A** 5-HT level in serum. **B** DA level in serum. **C** NE level in serum. Results are the mean ± standard error of the mean (*x* ± *s*), N = 6 per group. *P < 0.05, **P < 0.01, ***P < 0.001, compared to the control group. #*P* < 0.05, ##*P* < 0.01, ###*P* < 0.001, compared to the CUMS group

The 5-HT content in the CUMS group was significantly decreased compared to the control group (*P* = 0.0006), while the DA and NE contents showed a significant decrease (DA: *P* = 0.0004; NE: *P* = 0.0017) as well. FLX treatment significantly increased both 5-HT (*P* = 0.006) and DA contents (*P* = 0.0013), with a significant increase observed in NE content (*P* = 0.0331). The AP group exhibited a significant increase in 5-HT content (*P* = 0.0484), while there was an increase in DA and NE contents without reaching statistical significance; the EA group showed significant increases in both 5-HT (*P* = 0.0498) and DA contents (*P* = 0.0261), with an increase observed in NE content but not statistically significant. Although both AP and EA were able to improve the levels of these monoamine neurotransmitters, neither showed a significant advantage over the other. There were no significant differences observed between the Sham-AP group and the CUMS group regarding 5-HT, DA, and NE contents (Fig. 7).

## Discussion

Depression is a prevalent and pervasive disorder often accompanied by weight and appetite loss, anhedonia, reduced physical capacity, and other symptoms. The global burden of major depressive disorder (MDD) affects approximately 300 million individuals, with its incidence steadily rising over time. MDD has emerged as a leading cause of disability [31]. Currently, pharmacotherapy remains the primary approach for managing depression in clinical settings; however, antidepressants are constrained by their narrow target specificity, delayed onset of action, significant side effects profile, and potential for drug dependence [32]. Consequently, there is an imperative need to explore supplementary or alternative therapeutic modalities to address depression effectively.

According to traditional Chinese medicine theory, the etiology of depression may be attributed to disrupted Qi flow. Shangxing and Fengfu acupoints are well-established in treating mental disorders within traditional Chinese medicine, exhibiting superior therapeutic effects for depression. These acupoints are situated along the Du Mai meridian, which is closely associated with the brain and plays a crucial role in regulating Qi circulation throughout the body and modulating brain function [33]. Therefore, based on our previous experimental findings, we further investigated the underlying mechanisms through which acupuncture improves depressive-like behaviors in rats [34].

**Fig. 8:**
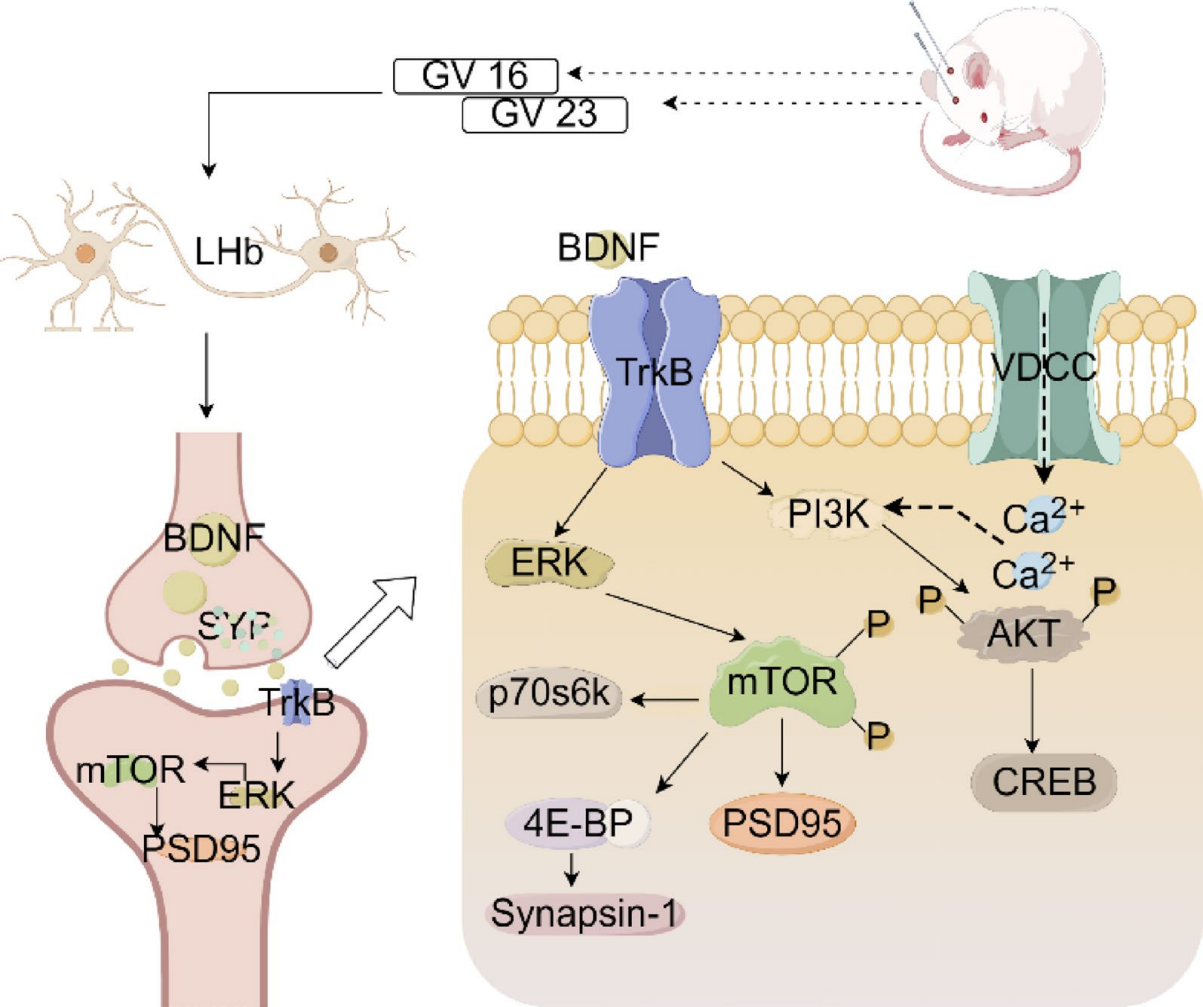
In the CUMS model, the schematic diagram illustrates the antidepressant mechanism of acupuncture. Acupuncture points Fengfu (GV16) and Shangxing (GV23) modulate the molecular mechanisms of synaptic plasticity associated with LHb, including the BDNF/ERK/mTOR signaling pathway and restoration of synaptic plasticity, thereby facilitating a reduction in depressive-like behaviors in rats

Currently, the specific etiology and pathogenesis of depression remain elusive. Various hypotheses, encompassing neurotransmitter transmission, neuroinflammation, genetic factors, oxidative stress, the role of neurotrophic factors, hypothalamic–pituitary–adrenal (HPA) axis dysfunction, stress response pathway dysregulation, and neuroplasticity have been implicated in the pathophysiology of depression [35]. Studies have demonstrated that depression not only impairs learning and memory but also disrupts brain structure and function by compromising synaptic plasticity [36]. Increasing evidence from studies on rodents, non-human primates, and humans suggests a correlation between abnormal activity in the lateral habenula and depressive symptoms [11]. Furthermore, synaptic plasticity changes within the lateral habenula have been implicated in developing long-term depressive symptoms [37]. Therefore, this experiment aims to investigate the effects of acupuncture on synaptic plasticity in the lateral habenula and its potential role in alleviating depressive-like behaviors in CUMS rats (Fig. 8).

The onset of depression is influenced by various factors, including psychological, genetic, social, and comorbid conditions [38]. Chronic Unpredictable Mild Stress (CUMS), a widely used animal model for depression, effectively replicates the chronic stress experienced by depressed patients and induces long-lasting depressive-like symptoms in animals that resemble human depression [39]. Behavioral experiments conducted in this study demonstrated that CUMS led to anhedonia, reduced motor and exploratory abilities, as well as behavioral despair in rats. Notably, both fluoxetine treatment and acupuncture significantly ameliorated the behavioral alterations induced by CUMS in rats. Furthermore, acupuncture also alleviated the weight gain deceleration caused by CUMS to varying degrees. These findings suggest that acupuncture may improve certain clinical manifestations of depression such as low mood, weight loss, and impaired motor ability.

BDNF can regulate synaptic plasticity within the brain [40]. As a crucial neurotrophic factor, BDNF plays a pivotal role in neuronal growth, development, and differentiation. The transmission of signals through BDNF and its receptor TrkB represents a key mechanism underlying the pathophysiology of depression as well as the therapeutic effects of antidepressants [41]. Studies have demonstrated that BDNF can induce antidepressant-like effects by enhancing synaptic plasticity in the CUMS mouse model [42]. In our current experiment, we observed reduced expression levels of both BDNF and its receptor TrkB in rats exposed to CUMS, suggesting an association between depressive-like behavior induced by CUMS and LHb-related BDNF signaling. This decrease was ameliorated by fluoxetine treatment and electroacupuncture. Furthermore, research has indicated that electroacupuncture may promote neuronal regeneration by increasing phosphorylation levels of cAMP response element-binding protein and elevating BDNF levels, thereby exerting antidepressant effects [43]. These findings suggest that acupuncture possesses potential for modulating brain neurotrophic factors and improving neural injury within the brain, which could play a significant role in depression treatment [44].

There is mounting evidence suggesting the crucial involvement of the mitogen-activated protein kinase (MAPK) pathway, specifically the extracellular signal-regulated kinase (ERK) subfamily, in the pathogenesis, symptoms, and treatment of depression [19]. Binding to TrkB, BDNF can activate its downstream ERK signaling pathway [45]. The ERK signalling pathway is vital in brain neural proliferation, differentiation, and neurogenesis and is essential for learning and memory functions. Studies have demonstrated that acupuncture may exert significant antidepressant-like effects on rats with depression induced by CUMS through modulation of the ERK signaling pathway [20]. Activation of the mTOR, which serves as a key downstream target for both Akt and ERK signal transduction pathways, represents an important therapeutic target for depression due to its regulation of cell growth, metabolism as well as coordination of neuronal signal transmission and excitability [46].

Moreover, previous studies have indicated that specific antidepressant medications can alleviate depressive symptoms by modulating the mTOR pathway. In our experiment, conducted on rats subjected to CUMS, the lateral raphe nucleus region exhibited significantly reduced phosphorylation levels within the ERK/mTOR signaling pathway, accompanied by downregulation in the expression of p-ERK and p-mTOR proteins. However, treatment with fluoxetine, acupuncture and electroacupuncture resulted in increased expression levels of these proteins, suggesting that acupuncture may exert its antidepressant effects through enhanced phosphorylation of the ERK/mTOR signaling pathway. The mTOR protein can form two distinct multi-component complexes: mTOR complex 1 (mTORC1) and mTOR complex 2 (mTORC2). Among these, mTORC1 can activate downstream protein targets such as p70 ribosomal S6 kinase (p70S6K) and eukaryotic initiation factor 4E binding protein 1 (4E-BP-1). Dysregulation in the mTORC1/p70S6K pathway has been implicated in the development of depressive behaviors [47–49]. In this study, CUMS significantly reduced the levels of phosphorylated p70S6K in the ventral tegmental area of rats, an effect that could be reversed by acupuncture. However, CUMS did not affect the expression of 4E-BP-1. These findings suggest that p70S6K, a downstream effector of the ERK/mTOR signaling pathway, may play a pivotal role in elucidating the potential mechanism underlying electrical stimulation for depression treatment.

SYP and PSD95 are localized on the presynaptic and postsynaptic membranes, respectively, playing crucial roles in regulating synaptic transmission efficiency and maintaining synaptic structural stability [50]. As hallmark proteins of these membranes, it has been reported that CUMS-induced depression in rat hippocampus leads to a reduction in dendritic spine density, neuronal count, as well as protein levels of MAP-2, PSD95, and SYP [51]. Dendritic spines are key sites for interneuronal synapses and are directly associated with neuronal structure and function. In our study, CUMS significantly downregulated the expression of SYP and PSD95. Fluoxetine significantly improved the expression of both SYP and PSD95, while acupuncture and electroacupuncture showed a more pronounced effect in enhancing SYP expression. Additionally, we observed a significant decrease in both number and density of dendritic spines following CUMS exposure; nevertheless, electroacupuncture treatment ameliorated this decline in dendritic spine density. These findings suggest a potential advantage of electrical stimulation over acupuncture in promoting dendritic spine recovery, but further studies with larger sample sizes are warranted to validate this difference. Therefore, we propose that the BDNF/ERK/mTOR signaling pathway may modulate synaptic plasticity within lateral habenula neurons while acupuncture exerts its antidepressant effects through this pathway.

Synapses are a fundamental functional component of the central nervous system, constantly influenced by external stimuli in daily life and impacting the brain’s structure and function [52]. Based on the findings of this experiment, it can be concluded that acupuncture can potentially enhance BDNF levels and its receptors, thereby activating the downstream ERK/mTOR signaling pathway and regulating synaptic plasticity to exert antidepressant effects. This study confirms that acupuncture can ameliorate depressive-like behaviors by modulating synaptic plasticity in the lateral habenula. Furthermore, acupuncture may also exhibit antidepressant effects through regulating synaptic plasticity or neurogenesis in other brain regions [53]. Relevant literature suggests that acupuncture possesses preventive properties against neuronal apoptosis, promotes synaptic plasticity, alleviates central and peripheral inflammatory responses, as well as regulates abnormalities in brain energy metabolism [54]. These findings provide additional possibilities for utilizing acupuncture in depression treatment and neurological disorders.

In this study, the sham acupuncture (Sham-AP) group received needling 3 mm lateral to the true acupoints GV16 and GV23, without manual manipulation or electrical stimulation. The absence of therapeutic effects in this group may be due to the deviation from the precise acupoint locations, which are believed to be essential for eliciting specific physiological responses. Additionally, the sham procedure did not produce the Deqi sensation, which is traditionally associated with effective acupuncture. These factors may have contributed to the lack of significant improvement observed in the sham-AP group.

Although both acupuncture and electroacupuncture showed therapeutic effects in alleviating depression-like behaviors in CUMS rats, the differences between the two interventions were not statistically significant. This may be attributed to several factors, including the similarity in acupoint selection and stimulation duration. Moreover, the intensity and frequency parameters used in electroacupuncture may not have been sufficiently optimized to yield a distinct advantage over acupuncture. Future studies should explore a broader range of electroacupuncture stimulation parameters, as well as investigate the underlying molecular and neurocircuitry differences between them, to further clarify their respective therapeutic mechanisms and potential differential efficacy.

Our study has several limitations that need to be considered. Firstly, although the sample size used in this study aligns with commonly accepted standards for exploratory preclinical research, it may limit the statistical power and the robustness of some molecular findings. We acknowledge this as a limitation and will incorporate larger sample sizes in future experiments to further validate and extend our current results. Secondly, although animal models provide valuable insights into mechanisms, they do not fully replicate the complexity of human depression. Including human subjects or advanced models could provide more relevant clinical insights. Moreover, our study mainly assessed outcomes at specific time points post-treatment, potentially missing the longer-term effects and fluctuations in treatment response over time. Longitudinal studies could provide a more comprehensive view of acupuncture’s therapeutic effects. Despite identifying the ERK/mTOR pathway as a potential target, the exact molecular mechanisms of acupuncture in depression remain unclear. Further research exploring additional pathways and targets could enhance our understanding.Methodologically, while we minimized biases, inherent experimental variations and environmental factors might have influenced outcomes.

## Conclusions

Our research shows that focused stimulation of the Fengfu (GV16) and Shangxing (GV23) acupuncture points effectively reduces depressive behaviors caused by CUMS in rats. This treatment’s effectiveness is based on activating the BDNF/ERK/mTOR signalling pathway in the lateral habenula, which encourages synaptic plasticity and triggers an antidepressant reaction. Specifically, acupuncture boosts the levels of key proteins and their phosphorylation in this pathway, which are crucial for neuronal adaptation and resistance against stress-induced depression. These details not only provide insight into the biological foundation of acupuncture’s therapeutic effects but also propose potential approaches for creating precise treatments for depression. Future translational research should investigate the practical relevance of these discoveries, potentially leading to new therapeutic strategies for treating depressive disorders.

## Data Availability

No datasets were generated or analysed during the current study.
